# Hallucinations Under Psychedelics and in the Schizophrenia Spectrum: An Interdisciplinary and Multiscale Comparison

**DOI:** 10.1093/schbul/sbaa117

**Published:** 2020-09-18

**Authors:** Pantelis Leptourgos, Martin Fortier-Davy, Robin Carhart-Harris, Philip R Corlett, David Dupuis, Adam L Halberstadt, Michael Kometer, Eva Kozakova, Frank LarØi, Tehseen N Noorani, Katrin H Preller, Flavie Waters, Yuliya Zaytseva, Renaud Jardri

**Affiliations:** 1 Department of Psychiatry, Connecticut Mental Health Center, Yale University, New Haven, CT; 2 Institut Jean Nicod, École des Hautes Études en Sciences Sociales, École Normale Supérieure, PSL Research University, Paris France; 3 Imperial College London, London, UK; 4 Department of Anthropology, University of Durham, Durham, UK; 5 Department of Psychiatry, University of California San Diego, La Jolla, CA; 6 Research Service, VA San Diego Healthcare System, San Diego, CA; 7 Pharmaco-Neuroimaging and Cognitive-Emotional Processing, Department of Psychiatry, Psychotherapy and Psychosomatics, Psychiatric University Hospital Zurich, University of Zurich, Zurich, Switzerland; 8 Department of Applied Neurosciences and Brain Imaging, National Institute of Mental Health, Klecany, Czechia; 9 Department of Psychology, Faculty of Arts, Charles University, Prague, Czechia; 10 Department of Biological and Medical Psychology, Faculty of Psychology, University of Bergen, Bergen, Norway; 11 Psychology and Neuroscience of Cognition Research Unit, University of Liège, Liège, Belgium; 12 Norwegian Center of Excellence for Mental Disorders Research, University of Oslo, Oslo, Norway; 13 School of Psychological Sciences, The University of Western Australia, Perth, Western Australia; 14 Department of Psychiatry and Medical Psychology, 3rd Faculty of Medicine, Charles University in Prague, Prague, Czechia; 15 Univ. Lille, INSERM U1172, CHU Lille, Lille Neuroscience & Cognition Centre (LiNC), Plasticity & SubjectivitY team, Lille, France; 16 Laboratoire de Neurosciences Cognitives et Computationnelles, ENS, INSERM U960, PSL Research University, Paris, France

**Keywords:** psychedelics, psychosis, hallucinations, serotonin, Bayesian, computational

## Abstract

The recent renaissance of psychedelic science has reignited interest in the similarity of drug-induced experiences to those more commonly observed in psychiatric contexts such as the schizophrenia-spectrum. This report from a multidisciplinary working group of the *International Consortium on Hallucinations Research* (ICHR) addresses this issue, putting special emphasis on hallucinatory experiences. We review evidence collected at different scales of understanding, from pharmacology to brain-imaging, phenomenology and anthropology, highlighting similarities and differences between hallucinations under psychedelics and in the schizophrenia-spectrum disorders. Finally, we attempt to integrate these findings using computational approaches and conclude with recommendations for future research.

## Introduction

Hallucinations, that is, percepts without corresponding stimulus, are common in psychiatric disorders (eg, schizophrenia spectrum disorders, a heterogeneous category with variable course and expressions; henceforth SCZs), in neurological disorders (eg, Parkinson’s disease, Lewy body dementia), while they can be observed in the general population too. They are also engendered by psychotomimetic drugs, including serotonergic agonists (ie, psychedelics). Since the nineteenth century, scientists have posited that clinical and pharmacological experiences could be related and that psychedelics might constitute a model of psychosis.^1^ The discovery of lysergic acid diethylamide (LSD) in 1943 was a boon to this “model psychosis theory,” spurring researchers to understand psychosis by administering psychedelics to healthy volunteers and by self-experimentation.^2,3^

The recent revival of psychedelic science generated new data and ideas, sparking great interest in the relevance of those compounds to psychosis. Do psychosis-related and drug-induced hallucinations share a similar etiology? Do they involve similar or overlapping neural mechanisms? How similar or different are these experiences phenomenologically and how are they each affected by culture?

This review from the *International Consortium on Hallucinations Research* (ICHR) aims to compare and contrast hallucinations under psychedelics with those observed in SCZs. Our working-group adopted a multiscale approach spanning multiple levels of understanding. First, we reviewed the underlying neural mechanisms, with a special focus on microscopic (synaptic) and macroscopic (network) mechanisms. Then, we described the subjective features of the two experiences, emphasizing their commonalities and differences and the impact of cultural factors. Finally, we described how computational models might connect these levels of analysis, from synapses to society.

## Pharmacology

At the synaptic level, SCZs has been linked to dopaminergic (DA) alterations, while classical psychedelic drugs, such as LSD, mescaline, and psilocybin, are serotonin (5-HT) receptor agonists. Psychedelics can be divided into three main structural classes: phenethylamines, tryptamines, and ergolines. The phenethylamines are relatively selective for 5-HT_2_ subtypes, whereas the tryptamines bind to a larger number of sites, including most 5-HT receptors and σ _1_ sites. Ergolines, by contrast, are even less selective and interact with serotonergic, dopaminergic, adrenergic, and histaminergic receptors. There is now a consensus that the 5-HT_2A_ receptor is the primary target for serotonergic hallucinogens in the brain. The first evidence linking the 5-HT_2A_ receptor to hallucinogenesis was derived from animal behavioral models (see table 1). For example, Glennon and colleagues found that 5-HT_2A_ antagonists, such as pirenperone and ketanserin, block the effects of psychedelics in drug discrimination (DD) studies conducted in rats.^4^ Those investigators also found that the potencies (ED_50_ values) of hallucinogens in the DD paradigm are robustly correlated with their 5-HT_2A_ affinity.^5^ The head-twitch response (HTR) assay is another behavioral paradigm that has been used in mechanistic studies of serotonergic hallucinogens. The HTR is a rapid reciprocal head movement that occurs in rodents after administration of serotonergic hallucinogens.^6,7^ Similar to the DD paradigm, selective 5-HT_2A_ receptor antagonists such as M100907 also block the HTR induced by hallucinogens.^8,9^ Likewise, LSD and other hallucinogens do not induce the HTR in 5-HT_2A_ knockout mice.^10,11^ The HTR paradigm has become increasingly popular in recent years because it is one of the few behavioral effects produced by hallucinogens that are not observed when animals are treated with non-hallucinogenic 5-HT_2A_ agonists such as lisuride, an LSD analog.^10,12,13^ There is also a robust correlation between the ED_50_ values of hallucinogens in the HTR paradigm and their potencies in humans and rat DD studies.^7^ Therefore, although the HTR assay does not directly model the psychedelic effects produced by hallucinogens, it serves as a behavioral readout of 5-HT_2A_ receptor activation that has considerable cross-species translational relevance.

**Table 1.** The pharmacology of psychedelics

	Drug Discrimination (DD)	Head-Twitch Response (HTR)	Prepulse Inhibition (PPI)	Exploratory and Investigatory Behavior
Behavioral effect	Rats can be trained to discriminate hallucinogens from vehicle.	Rats and mice treated with hallucinogens express the HTR.	Hallucinogens reduce PPI in rats.	Hallucinogens reduce exploratory and investigatory behavior in rats.
Receptor mechanism for phenylalkylamine hallucinogens (eg, mescaline)	Selective 5-HT_2A_ receptor antagonists (eg, M100907 and MDL 11,939) block the effect.	Selective 5-HT_2A_ receptor antagonists (eg, M100907 and MDL 11,939) block the effect.	Selective 5-HT_2A_ receptor antagonists (eg, M100907 and MDL 11,939) block the effect.	Selective 5-HT_2A_ receptor antagonists (eg, M100907 and MDL 11,939) block the effect.
Receptor mechanism for indoleamine hallucinogens (eg, LSD and psilocybin)	The mechanism for the DD effects for tryptamine hallucinogens often mediated by both 5-HT_1A_ and 5-HT_2A_ receptors. The mechanism for the effect of LSD is time-dependent; at short intervals between injection and testing (eg, 15–30 min), the effect of LSD is blocked by selective 5-HT_2A_ antagonists; however, if the interval is increased to 90 min then the effect of LSD is blocked by antagonists of D_2_-like receptors.	Selective 5-HT_2A_ receptor antagonists (eg, M100907 and MDL 11,939) block the effect.	Selective 5-HT_2A_ receptor antagonists (eg, M100907 and MDL 11,939) block the effect.	Indoleamine hallucinogens act through both 5-HT_1A_ and 5-HT_2A_ receptor mechanisms.
Sensitivity to lisuride	Lisuride produces hallucinogen-like effects in some DD studies but not in others.	Lisuride does not induce the HTR in rats or mice.	Lisuride reduces PPI in rats but the effect is mediated by D_2/3_ receptors rather than the 5-HT_2A_ receptor.	Lisuride does not produce LSD-like effects on exploratory or investigatory behavior in rats.

In addition to DD and HTR, several other behavioral paradigms are commonly used to study the effects and pharmacology of hallucinogens in rodents. Prepulse inhibition (PPI) of the startle reflex is one example. PPI refers to the phenomenon where a weak prestimulus will inhibit the response to a subsequent startle-inducing pulse. This effect is commonly used as an operational measure of sensorimotor gating. LSD and other hallucinogens inhibit PPI in rats, an effect that can be blocked by pretreatment with selective 5-HT_2A_ receptor antagonists (eg, M100907 and MDL 11,939).^11,14^ Although lisuride also reduces PPI in rats, its effect is blocked by DA D_2/3_ receptor antagonists but not by MDL 11,939. Similar findings have also emerged from studies of exploratory behavior in rats. Although hallucinogens reduce exploratory locomotor activity in a novel environment via 5-HT_2A_ receptor activation,^15,16^ lisuride produces a qualitatively different behavioral profile similar to the effect of DA receptor agonists.^17^ Hallucinogens also alter timing behavior in rats and mice via 5-HT_2A_ receptor activation.^18–20^

Although the 5-HT_2A_ receptor was first linked to the mechanism of action of hallucinogens in 1984, it took more than a decade to generate relevant evidence in humans. In 1998, a clinical study conducted by Franz Vollenweider and colleagues confirmed that ketanserin can block the subjective effects of psilocybin.^21^ The 5-HT_2A_/D_2_ receptor antagonist risperidone can also block the subjective response to psilocybin, whereas the D_2_ antagonist haloperidol was not effective.^21^ More recently, similar findings were reported for LSD. Although there has been speculation that D_2_ receptor activation may contribute to the psychopharmacology of LSD, ketanserin seems to have little effect on D_2_ sites but is capable of blocking the subjective and neural response to LSD.^22–24^ Notably, it was also reported recently that the intensity of the subjective response to psilocybin is correlated with the level of central 5-HT_2A_ receptor occupancy.^25^

## Brain-Imaging Markers

At the network level, SCZs and psychedelics exhibit interesting commonalities and differences. A first line of work comes from fMRI capture studies which compare ON and OFF periods for hallucinations and detect the phasic neural changes associated with hallucinatory ON states. In SCZs, these studies suggest a role for modality-dependent associative cortex overactivations during hallucinations.^26–30^ When recruited, the primary cortices were associated with more vivid experiences.^28^ Interestingly, the onset of hallucinations has been found associated with various aberrant activation/deactivation patterns. Hyperactivity was found in the hippocampal complex, as well as within associative cortices related to the hallucinatory content, while the default-mode network was found concomitantly deactivated.^28,31^

Brain imaging studies conducted to explore psychedelic states did not try to specifically capture hallucinatory events, but rather focused on neural changes in relation to sensory experiences during the psychedelic intoxication, making links with hallucinations more indirect. Regarding visual hallucinations (VH), a greater cerebral blood flow was measured in the visual cortex under LSD.^32^ Increased early visual activity but decreased processing in associative visual areas was also observed after psilocybin administration,^33^ suggesting that a combination of enhanced early sensory and reduced associative processing may contribute to the psychedelic experience.^23,34^

The second contribution comes from large-scale neural connectivity analyses, based on functional connectivity (FC; correlations between signals measured in different brain areas that define intrinsic brain networks), and effective connectivity, namely the effect one neuronal system exerts over another. We first look at FC studies and then briefly look at selective changes in directed effective connectivity.

A well-replicated finding in healthy individuals is an antagonistic activity between the default-mode resting-state network (DMN) and the task-related central-executive network (CEN).^35–40^ Some authors proposed that the orthogonality of these networks might break down in psychotic states.^41^ A functional disconnection between the nodes of the DMN and CEN might notably engender impaired self-monitoring as observed in SCZs^42^ and manifest as weak anti-correlation between these intrinsic brain networks.

According to the triple-network theory,^43^ the antagonistic activity of these resting-state networks (DMN and CEN) putatively reflects competing modes of information processing that may be regulated by the salience network (SN).^44^ Recent experimental data using intracranial EEG reported temporal profiles of task-evoked activity compatible with the hypothesis of SN acting as a switch between the CEN and DMN.^45^ Impairments of the triple-network was proposed broadly involved in psychopathology,^46–48^ and more specifically in intrusive experiences, such as flash-backs,^49^ obsessive ideas,^50^ or hallucinations in SCZs.^31^ In this vein, it has been proposed that SN impairments may reflect a disturbance in ascribing salience properly,^51^ while DMN instabilities seem to be a shared characteristic across multiple sensory domains in patients with hallucinations.^41^

Classical psychedelics also induce pervasive changes in network-dynamics that can generally be described as a transition from regularity to increased instability. The coherence of classical resting-state networks was found diminished (disintegrated), while FC of the primary visual cortex expanded—desegregated.^32,52^ In complement to its reduced activity-level, the DMN was found to potentially co-activate with the CEN, a phenomenon which may underlie the reported confusion between internally and externally generated mental contents.^53^ Analyzing global brain connectivity with fMRI after the administration of LSD and psilocybin also revealed an increased integration of sensory and somatomotor information together with a disintegration of information from associative networks.^23,54^ Additionally, a general decrease in directed FC, and concurrently an increase in undirected FC after the administration of LSD was observed using MEG imaging and may point to increased instability in psychedelic states.^55^

Another influential theory in SCZs is the thalamic filter hypothesis (wherein the thalamus gates sensory information to prevent the information overflow in the cortex^56^). Resting-state fMRI studies in patients at various stages of the illness showed that prefrontal–thalamic FC was decreased, while thalamic FC with somatosensory and motor areas was strengthened during disease progression, in a manner that correlates with positive symptoms.^57,58^ However, findings regarding the exact relationship between thalamocortical dysconnectivity and clinical symptoms are mixed.^59^

Thalamocortical connectivity was found altered in psychedelic states. Specifically, LSD was found to selectively increase effective connectivity from the thalamus to certain DMN areas, while other connections are attenuated.^60^ Furthermore, increased thalamic connectivity with the right fusiform gyrus and the anterior insula correlated with visual and auditory hallucinations (AH), respectively.^61^

In summary (see table 2), hallucinations relate more to associative network overactivations in SCZs, while they are linked with primary cortex overactivations under psychedelics. Second, in both cases, the experience is associated with reduced internal integration of functional networks, an enhanced correlation between internally and externally oriented networks as well as an impaired thalamocortical connectivity. This phenomenon may notably blur the differentiation between self-generated and perceived mental contents.

**Table 2.** Comparison of the brain-imaging markers of psychotic and serotonergic hallucinations

	Schizophrenia Spectrum	5-HT_2A_ Agonists	Comparison
Major networks a)During rest b)During task	a) DMN hypoactivation Decreased connectivity within and (mostly) between RSN b) Lack of DMN suppression during tasks—decreased DMN and CEN anticorrelation	a) DMN hypoactivation Decreased connectivity within—increased connectivity between RSN Decreased DMN and CEN anticorrelation b) Not enough evidence	a) Partially similar Differences in changes in connectivity between RSN
Hallucinations a) Symptom capture b) Resting-state analysis	a) Activation of hippocampus and modality-specific secondary cortex with deactivation of DMN and activation of SN and CEN b) Thalamic connectivity with prefrontal cortex lowered—thalamic connectivity with somatosensory cortex increased	a) Not available b) Increased activity in primary visual areas—decreased activity in associative areas Preserved thalamic connectivity with DMN—increased thalamic connectivity with CEN	a) Directly incomparable—mostly primary cortices in psychedelics—mostly associative cortices in SCZs
Link with experience	AH altered resting-state connectivity in left temporal areas VH increased RS connectivity between visual cortex and amygdala in SCZs (AH and VH)	VH / imagery expanded connectivity and activity of V1—VH/imagery correlated with CEN activation	

RSNs, resting-state networks; DMN, default mode network; CEN, central-executive network; SN, salience network.

## Phenomenology

In terms of the sensory modalities involved, AH are the most common modality of hallucinations in SCZs, with a prevalence of around 79%.^62^ AH are three times as frequent as VH, which have a mean prevalence of approximately 27%.^62,63^ The exact prevalence of hallucinations in other modalities is largely unknown, with significant variation between studies. Estimates vary for olfactory hallucinations (6–26%), gustatory hallucinations (1–31%), and somatic or tactile hallucinations (4–19%).^62,64,65^ AH occur alone approximately half of the time,^66,67^ while hallucinations in other modalities almost never occur alone.^66,68^ Some studies report that multimodal or “fused” hallucinations (MMH; eg, seeing a talking head)^69^ are highly prevalent in SCZs,^65,70–72^ whereas other reports suggest that these hallucinations are rare.^73^ By contrast, hallucinations induced by 5-HT_2A_ agonists occur primarily in the visual domain^74^ (a shared feature with neurological disorders, such as Parkinson’s disease and Lewy body dementia). Nevertheless, distortions of body image, tactile hallucinations, and auditory alterations are not uncommon, especially when hallucinogens such as DMT or psilocybin are administered at high doses.^75–77^ Audio-visual experiences have frequently been reported, but whether they qualify as hallucinations (or synesthesias) is still debated.^77^ Olfactory and gustatory hallucinations are very rare in comparison, but have occasionally been reported.^78^ Synesthesia-like experiences are also very common with serotonergic hallucinogens^79^ but are uncommon in SCZs.^63^

With respect to the content of VH, serotonergic hallucinogens induce both elementary (brightly colored geometric form constants such as lattices, cobwebs, tunnels, and spirals)^78^ and complex hallucinations.^74,76,80^ Complex hallucinations are images of scenes or landscapes, often containing “ordinary” (humans, animals, artifacts, etc.) and “extraordinary” entities (chimeras, spirits, aliens, monsters, etc.). The prevalence of complex hallucinations increases with drug dose^76,81^ and as the psychedelic experience progresses over time.^82^ In SCZs, VH more often includes life-size images of faces, people, objects, or events, which may be bizarre or frightening. Typically, the hallucinations experienced in SCZs are detailed, concrete, and well-anchored in space.^83^

A series of experiential changes often precede the onset of psychosis, including AH (for a review, see Refs. ^84,85^). The occurrence of these prodromal hallucinations often provokes intense emotions; they may be attributed to a supernatural origin and viewed as a sign of a larger meaning or fate.^86^ Similarly, the VH induced by 5-HT_2A_ agonists are often very meaningful and can be imbued with strong existential, metaphysical, and religious overtones.^80,87–89^

Psychosis is often accompanied by very rich and detailed hallucinations that are experienced as vivid, real, and beyond volitional control.^83,90^ There may be profound changes in attention, reality testing, and memory.^91^ Although the hallucinations induced by 5-HT_2A_ agonists can be extremely vivid and may even feel more real than everyday sensory experiences, insight about their etiology is typically preserved; in other words, reality testing is not impaired and subjects using hallucinogens can typically distinguish between drug effects and normal waking consciousness.^80,92,93^ In contrast, in SCZs, hallucinations tend to be more difficult to discriminate from every-day perception. An important contributing factor is the contextual differences between the two states: while psychotic episodes in SCZs occur recurrently and unpredictably, the psychedelic state is transient (the nature and prevalence of chronic perceptual abnormalities, such as acid flashbacks and the hallucinogen persisting perceptual disorder are still debated^77,94^), purposeful and voluntarily initiated, thus marked by a special sense of agency (see Anthropology section).

As summarized in table 3, psychotic and serotonergic hallucinations differ in many respects: most notably in the modalities involved in the types of hallucinatory objects, and in the reality status ascribed to hallucinations. Yet, some commonalities can also be identified, especially as regards the meaningfulness, the emotional significance, and the metaphysical/spiritual quality of hallucinations (cf. Ref. ^95^).

**Table 3.** Comparison of the phenomenology of psychotic and serotonergic hallucinations

	Schizophrenia Spectrum	5-HT_2A_ Agonists	Comparison
Sensory modalities	Mainly AH (multimodal in some cases)	Mainly VH (multimodal in some cases)	Different
Content	No geometric hallucinations Complex hallucinations (mostly ordinary entities)	Geometric hallucinations Complex hallucinations (ordinary and extraordinary entities)	Different
Meaning	Strong existential/metaphysical meaning	Strong existential/metaphysical meaning	Similar
Reality monitoring/insight	Poor reality monitoring and insight	Reality monitoring and insight often preserved	Different
Duration	Recurrent psychotic episodes; they can last from several weeks to several months. Hallucinatory episodes during psychotic episodes can last several seconds or minutes; continuously present in some individuals.	Transient states, lasting a few hours. Long-term perceptual effects are rare.	Different

## Anthropology

Both in relation to psychedelic use and SCZs pathology, anthropological studies reveal enormous cultural variation that would benefit from a more systematic study. Comparative anthropological studies show that some features of the experiences induced by hallucinogenic plants and mushrooms are similar across cultures (eg, geometric VH), while others vary extensively cross-culturally (eg, subjective feeling tone, meaning, or content of the hallucinations).^96–98^ Hallucinogenic substances such as serotonergic plants and mushrooms have been traditionally employed in a variety of sociocultural purposes. For example, species of *Anadenanthera* and *Virola*, psilocybin mushrooms, and peyote have been used for divinatory and healing purposes.^99–106^ Some of these plants have also been employed in initiation rituals.^107,108^ It is worth highlighting that these hallucinogens have also traditionally been used for “non-ritualistic” purposes, for instance, in warfare^109,110^ and hunting.^111^ Finally, as illustrated by the case of “psilocybin mushrooms parties” held in Mexico, the pre-Columbian recreational use of these plants has been documented.^112^

Observing homogeneity in the features of the hallucinations produced by psychedelics within the same culture, many ethnographers have defended a culturalist approach to psychedelic hallucinations.^97,107,113–117^ For instance, terms such as “culturally influenced visions” ^117^ or “stereotypic visions” ^97^ have been used to argue that cultural variables are significant in shaping the hallucinogenic experience. Several candidates have been proposed to shed light on the vectors of this enculturation of the hallucinatory content: mythological and cosmological knowledge,^118^ kinship system and gender,^118^ iconographic representations,^117^ verbal exchanges and ritual interactions.^119,120^ However, these factors, the underpinnings of their effectiveness, and the sensitivity of different psychedelic substances to their effects require further study.

In the laboratory context, there have been few attempts to identify and experimentally manipulate nondrug variables in studies of serotonergic psychedelics (see Refs. ^121,122^ for an overview). In one exception, Studerus et al^123^ analyzed data from 23 controlled experimental studies, concluding that: the personality trait of absorption (“openness to cognitive, perceptual, imagistic, and other experiences”), the state of mind immediately prior to drug intake and having had few psychological problems in the prior weeks, were most strongly associated with positive experiences, while emotional excitability, young age, and an equipment-heavy experimental setting, were most strongly associated with negative experiences. In the resurgence of clinical therapeutics, extra-pharmacological variables considered especially important for therapeutic outcomes include a safe and supported treatment space, bespoke therapeutic support from trusted guides and appropriate music to accompany psychedelic sessions.^124–126^

There has also been little cross-cultural research on variability in hallucinations in SCZs. Nuevo et al^127^ conducted a cross-country study of prevalence of hallucinations finding high variability (eg, from 0.8% in Vietnam to 31.4% in Nepal), but did not analyze this further in order to uncover any potential cross-cultural patterns or correlations between specific cultural factors and the phenomenology of hallucinations. Luhrmann et al^128^ compared AH in SCZs patients in the United States, India, and Ghana, arguing that the negative content of AH varied according to culture. However, this was a qualitative, interview-based study, with small numbers, and groups were not compared or matched in terms of co-attendant clinical variables. A large number of questions remain unanswered in terms of what role culture may play in shaping hallucinations (for more detail, see Ref. ^129^). The relationship between hallucinations and culture in SCZs and in the use of psychedelics, and the possible overlap between these two research areas merits further study, not least because techniques traditionally mobilized to shape the phenomenology of psychedelic hallucinations in native societies in the Americas may enrich the therapeutic engagement with hallucinations in non-native contexts.^130^ This could be especially useful in cases where hallucinations respond minimally to antipsychotic medication.

## Computational Modeling

In previous sections, we described psychedelic experiences and contrasted them with psychotic experiences in SCZs. We notably focused on the potential neural mechanisms that may support those experiences, both at the level of synapses (pharmacology) and networks (brain-imaging). Then, we explored the first-person experience (phenomenology) and described how it can be shaped by the social and cultural milieu (anthropology). Despite such a multi-scale approach, our endeavor would be incomplete without discussing the links between them. Besides, another relevant question remains open: Could hallucinations with different phenomenology and neurobiology be underlain by (partially) similar mechanisms? To address those questions, we turn to the burgeoning field of computational psychiatry^131^ and discuss how information processing might hold the key to both answers.

Computational models conceive the brain as an information processing system and provide normative accounts of those processes, which are then mapped onto existing neural structures.^131^ We will focus on one particular type of computational models: Bayesian models.^132,133^ The main idea behind this framework is that the brain learns generative models, that is, internal, hierarchical representations of the causal structure of the world.^134,135^ When new inputs enter the system through the sensors, they are combined with prior information (accumulated knowledge which might include expectations, memories, etc.) to generate predictions about the causes of the sensory input. In short, Bayesian models conceptualize the brain as an inference machine that tests multiple hypotheses about the state of the world, the body or the brain itself and picks the most probable one. We will summarize Bayesian theories that situate the synaptic disconnections implicit in the neuropharmacology of psychedelics (and hallucinations) in the larger context of abnormal functional and effective connectivity studies reviewed above. The basic premise rests on linking false (perceptual) inference to disconnections or disintegration of the psyche (in the sense of Bleuler), conceiving of hallucinations as aberrant perceptual inference due to abnormal belief updating, particularly in terms of how abnormal synaptic connectivity can lead to false inference via inappropriate weighting of sensory evidence and prior beliefs. This inappropriate weighting, via neuromodulation, could underwrite hallucinations in both SCZs and psychedelic states.

**Fig. 1.**
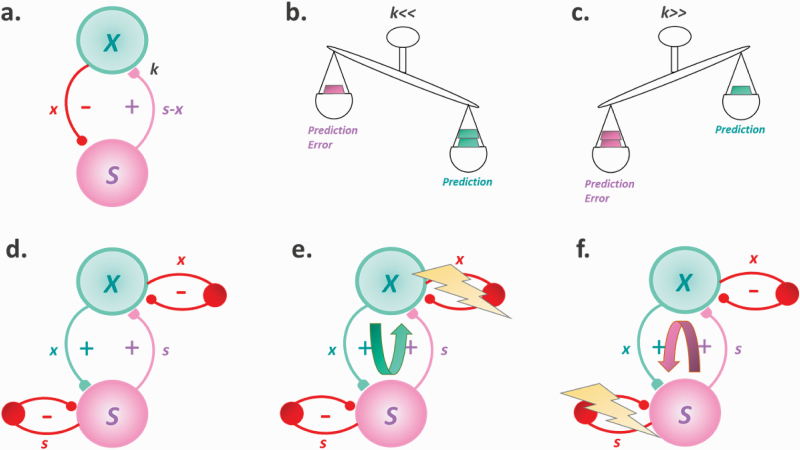
Illustration of different Bayesian models of hallucinations. (a–c) The predictive coding framework. (d–f) The circular inference framework. X, hidden cause; S, sensory variable; x and s, predictions and sensory messages; s-x, prediction error; k, relative weight of inputs as compared to predictions (Kalman gain).

Inference can be implemented in various ways. According to predictive coding^136^ (ie, Kalman filtering^137,138^ or variational free-energy minimization^139^), new sensory inputs are constantly explained-away by inhibitory feedback signals (sent from higher level areas to lower level areas, that “modulate” sensory inputs according to the behavioral context; ie, predictions; figure 1a). When predictions cannot fully explain the input, a residual error-signal (ie, prediction error [PE]) is sent up in the hierarchy to update the dominant hypothesis (belief), thereby reducing surprise (or surprisal). Conversely, when predictions and inputs match, no PE is generated and thus, the current model is sustained. It is worth noting that, under certain formulations, surprise can also be minimized by appropriate action (active sampling of the environment, ie, active inference^140^), also explaining exploratory behavior and long-term minimization of PE.^141^ Crucially, both predictions and inputs are weighted according to their reliability (parameter *k* in figure 1; Kalman gain), resulting in precision-weighted PE. In one of the first articles to suggest a computational account of psychedelics, Corlett and colleagues suggested that psychedelics act by increasing the prior weight (thus decreasing *k*), which results in inferences being mainly driven by expectations (figure 1b).^142^ The group also suggested a tentative neural mechanism for this prior overweighting, namely “excessive AMPA-receptor signaling, in the absence of NMDA-receptor impairment.” Importantly, it has been argued that the same mechanism might underlie hallucinations in SCZs,^143–145^ with a recent study validating this theory and, additionally, providing evidence for over-weighted priors in a group of nonclinical voice hearers.^146^ Taken together, those theories and evidence suggest that hallucinations might reflect the same underlying computational mechanism, regardless of the exhibited phenomenology or clinical context.

The idea that serotonergic agonists increase prior weight is not unanimously accepted. In a recent article, Carhart-Harris and Friston suggested that the opposite might also be true, namely a relaxation of the priors that increases *k* (figure 1c).^147^ Their REBUS theory explains, among other things, the potential therapeutic effects of psychedelics (eg, in depressive disorders), mediated by a relaxation of pathological priors associated with those illnesses. Intriguingly, although the REBUS and the strong-prior theory seem at first sight incompatible, this is not necessarily true. In particular, priors, can be both over- and under-weighted, but at different levels in the cortical hierarchy, for example, weak low-level priors (high *k*) might be compensated by stronger high-level priors (low *k*).^148^

Although predictive coding is a powerful inference scheme, it is not the only one. For example, one could replace inhibitory priors with excitatory priors, resulting in a closely related algorithm in which beliefs are not updated by error-signals, but by the sensory inputs per se (Belief Propagation [BP]; figure 1d). Despite its generality and simplicity, BP postulates recurrent, excitatory connections. Without well-tuned control mechanisms (eg, inhibitory control), it results in information loops, a form of “run-away excitation” where beliefs are erroneously amplified and the feed-forward (input) and feedback (prediction) messages become aberrantly correlated (Circular Inference^149,150^). There are two types of loops: descending (overcounted priors; figure 1e) and ascending (overcounted inputs; figure 1f). Importantly, different loops result in different types of aberrant percepts: while ascending loops induce unimodal hallucinations (eg, AH in SCZs), descending loops give rise to multisensory phenomena (eg, synesthesia-like experiences; MMH induced by DMT).^151^ Although the former link between ascending loops and SCZs has already been empirically established,^152^ the latter between descending loops and psychedelics remains purely theoretical and still needs experimental support.

## Conclusion

In this article, we sought to compare and contrast hallucinations in SCZs and under psychedelics. We identified several interesting common features: both experiences are related to a reduced integration and stability of functional networks, as well as a distorted anti-correlation between resting-state and task-positive networks. Furthermore, both experiences are afforded a strong metaphysical meaning. We also highlighted various crucial differences: First, psychedelics over-engage primary sensory cortices, hallucinations in SCZs, on the other hand, are mostly related to overactivation of associative networks. Furthermore, while drug-induced psychosis mostly encompasses VH (often geometric) with preserved insight, SCZs is characterized by AH (mostly voices) and poor reality monitoring. Additionally, we pointed out a number of topics that need further investigation, more particularly the role of serotonin in SCZs, the prevalence of MMH in both experiences and the potential cultural impact on hallucinations in SCZs. Finally, we suggested that psychotic experiences, regardless of their diagnostic categorization, might be underlain by the same computational mechanisms that tie together subjectivity and neural implementation, namely altered predictive processing. Future studies will have to clarify whether the same (eg, strong priors) or different (eg, climbing vs descending loops) impairments underscore these different psychotic experiences.
